# Correlation between safety attitudes and early adoption of cognitive aids in the German culture sphere: a multicenter survey study

**DOI:** 10.1186/s12913-022-08581-3

**Published:** 2022-09-30

**Authors:** Justus Wegener, Michael St.Pierre, Oliver Keil, Hendrik Eismann

**Affiliations:** 1 Business School, Middlesex University London, The Burroughs, London, NW4 4BT UK; 2 Anästhesiologische Klinik, Universitätsklinikum Erlangen, Krankenhaustrasse 12, 91054 Erlangen, Germany; 3 Department of Anaesthesiology and Intensive Care Medicine, Hannover Medical School, Carl-Neuberg-Str. 1, 30625 Hannover, Germany

**Keywords:** Cognitive Aid implementation, Checklist implementation, Safety Attitudes Questionnaire (SAQ), Elektronische Gedächtnis- und Entscheidungshilfe für Notfälle in der Anästhesiologie (eGENA)

## Abstract

**Background:**

Cognitive Aids (checklists) are a common tool to improve patient safety. But the factors for their successful implementation and continuous use are not yet fully understood. Recent publications suggest safety culture to play a key role in this context. However, the effects on the outcome of implementation measures remain unclear. Hospitals and clinics that are involved in cognitive aid development and research might have significantly different safety cultures than their counterparts, resulting in skewed assessments of proper implementation. Therefore, the objective of this study was to assess the correlation between cognitive aid implementation and safety attitudes of staff members in early adopting and later adopting clinics.

**Methods:**

An online survey of the Safety Attitudes Questionnaire (SAQ) was carried out in German anaesthesiology departments during the initial implementation of a new checklist for emergencies during anesthesia (“eGENA” app). Subsequently an analysis between subgroups (“eGENA” app usage and occupation), with Kruskal–Wallis- and Mann–Whitney-U-Tests was carried out for the general SAQ, as well as it six subscales.

**Results:**

Departments that introduced “eGENA” app (Median 3,74, IQR 0,90) reported a significantly higher median SAQ (U (N_eGENA_ = 6, N_non eGENA_ = 14) = 70,0, z = 2,31, *p* = 0,02, *r* = 0,516) than their counterparts (Median 2,82, IQR 0,77) with significant differences in the dimensions teamwork climate, work satisfaction, perception of management and working conditions.

**Conclusion:**

Early adopters of cognitive aids are likely to show a significantly higher perception of safety culture in the SAQ. Consequently, successful implementation steps from these settings might not be sufficient in different clinics. Therefore, further investigation of the effects of safety culture on cognitive aid implementation should be conducted.

## Introduction

Since Kohn’s landmark publication “To err is human” [1] patient safety became a growing concern and research topic in the last two decades. Several strategies and concepts for improvement have been developed and implemented. One of these are cognitive aids (e.g. checklists, medication dosing charts). Generally their aim is to reduce the risk of errors associated with high or low levels of cognitive strain [2]. Their effects have been researched globally, showing a wide range of results, mostly supporting their use. Adequately designed and implemented cognitive aids have repeatedly, in different contexts, proven to significantly improve clinically relevant measurements including a reduction in mortality and complications [3] as well as staff centered measurements such as improving handover quality [3] and staff satisfaction [4]. In spite of their low costs and success in other industries (e.g. aviation), implementation of cognitive aids in healthcare settings still has to overcome specific challenges [5] resulting in heterogenic quality of their design and commonness of their application.

Because of their benefits policymakers increasingly start to include cognitive aids in their considerations and guidelines [6]. But these tools also have inherent risks (e.g. checklist fatigue) caused by improper implementation and design [7], as well as significant opportunity costs [8]. This results in complex management challenges for healthcare leaders. While some research into the implementation of cognitive aids has already been conducted, the factors for the success of implementation are not yet fully understood [9]. Safety culture repeatedly has been shown to be both a significant facilitator and hurdle for implementation success [10, 11]. While several studies have shown an increase in measurements of safety culture during implementation measures [12], differences between these early adopters and innovators and later adopting institutions has not been explored. But these differences might inhibit successful implementation processes in later adopting organizations. Consequently, this study investigates the interrelation between cognitive aid implementation and safety culture during the initial implementation of an electronic cognitive aid for crisis management in anesthesia (eGENA) through a multicenter survey in early and later adopting departments using the Safety Attitudes Questionnaire (SAQ).

Some studies into safety culture have found a significant difference in safety culture between different occupations in the same departments [13]. Since these tools are most commonly used interprofessionally, and therefore rely on adoption by all occupations, a significant impact on cognitive aid implementation might stem from these differences. Therefore, this study aims to replicate differences between occupations.

## Methods

### Survey instrument

Safety culture is generally understood as a multidimensional concept, with an ongoing debate of its definition. Some authors distinguish between safety culture and safety climate, while others use the term synonymously [14]. Because of its importance not only in healthcare but also in other industries (e.g. aviation, nuclear) several instruments have been developed for its quantification [14]. Of these the Safety and Attitudes Questionnaire (SAQ) has been used most commonly in the context of cognitive aids in healthcare [15–18]. Originally developed as the Flight Management Attitudes Questionnaire to asses safety culture in aviation, multiple versions of the SAQ have been developed for different clinical surroundings (e.g. intensive care, operating rooms and inpatient settings) [19]. These all contain 30 unchanged core items measuring six dimensions of safety culture (teamwork climate, safety climate, job satisfaction, stress recognition, perception of management and working conditions). Zimmermann et al. have translated these into German and published a validation study, with satisfactory psychometric properties in all dimension but perception of management [20].

### Data collection

Since emergencies during anesthesia pose a significant challenge in terms of decision making and team management the use of cognitive aids seems reasonable [10, 21], the German Society of Anaesthesia and Intensive Care (DGAI) and the German Professional Association of Anaesthetists (BDA) decided to implement the German Cognitive Aid Working Group with the aim of develop a Cognitive Aid for crisis management in anesthesia. The working group developed an electronic cognitive aid called eGENA (elektronische Gedächtnis- und Entscheidungshilfe für Notfälle in der Anästhesiologie) [2] applying a user centered design process and a multistep implementation strategy. The working group developed not only the actual cognitive aid but also an editor, which can be used to adapt the checklist to the local context, as well as a training concept to adequately train staff [22]. After the development several papers describing the design process, the functionality, as well as recommended steps for its implementation have been published [2, 22–25]. Currently, however, it is not mandatory for German anaesthesia departments to use the cognitive aid during intraoperative emergencies. Although the cognitive aid is distributed for free and can be downloaded without prior registration, departments that were interested in adopting the cognitive aid were encouraged to contact the working group for further information and support.

During the initial implementation phase some of these departments already adopted eGENA (early adopters) while others were still in various stages of planning or decided against implementation (late adopters). We reached out to twenty-five departments, that had inquired about eGENA in the past. All departments contacted were departments or clinics for anaesthesiology (some had an additional focus on critical care medicine). They agreed to be contacted by the German Cognitive Aid Working Group for research purposes and were asked to participate in an online survey (SoSci Survey Version 3.2.12 (Germany)). The survey entailed questions about the usage of eGENA at the participant’s hospitals, as well as the German language version of the SAQ (SAQ-GER) by Zimmermann et al. and sociodemographic questions (occupation, gender and relevant work experience). The survey was accessible from February until June 2021. Since a consecutive increase of measurements of safety culture after cognitive aid implementation has been demonstrated [12] and as we expected low response rates due to high workload in German anaesthesia departments during the COVID-19-Pandemic we opted for a single survey period.

### Statistical analysis

Statistical analysis was carried out with IBM SPSS Statistics Version 28.0.0.0. (USA). Since the scale “perception of management” has not yet shown sufficient internal validity in the SAQ-GER we evaluated Cronbach’s ⍺ for the SAQ und it’s subscales, while accepting values between 0,70 and 0,95. To determine adequate selection of tests for comparing subgroups, both the Kolmogorov–Smirnov- and Shapiro–Wilk-test were performed assuming normal distribution for *p* > 0,05. Subsequently, an analysis between the subgroups was carried out with the Kruskal–Wallis- as well as the Mann–Whitney-U-test.

## Results

Of the twenty-five departments contacted, seven agreed to participate in the study. After the end of the survey period 24 data sets could be collected. The respondents were predominantly male (58,3%), use eGENA in their institution (62,5%) and work as physicians (58,3%). Table 1 shows the profession and the relevant work experience of the respondents.

**Table 1:** Occupation and relevant work experience of the respondents

**Occupation**	***N* = 24**	**%**
** Nurses**	10	41,6
Anaesthesia nurse	2	8,3
Specialised anasthesia nurse	5	20,8
Anaesthesia technician	3	12,5
** Physicians**	14	58,3
Residents	6	25
Consultant	8	33,3
**Relevant work experience**	***N* = 24**	**%**
< 2 Years	5	20,8
2–5 Years	3	12,5
5–10 Years	5	20,8
10–15 Years	5	20,8
> 15 Years	6	25

The evaluation of Cronbach’s alpha showed sufficient internal validity with 0,923 for the SAQ (30 Items). This did not persist for the subscale “perception of management” with a value of 0,366 (4 Items). Further analysis showed that the Item “management does not knowingly compromise the safety of patients” had a negative inter-item-correlation with -0,596 which was therefore excluded from further analysis, resulting in sufficient internal validity in the SAQ and all subscales (Table 2).

**Table 2:** Cronbach's ⍺ of SAQ-Scales

Scale	Cronbach’s ⍺	Items
Teamwork climate	0,832	6
Safety climate	0,780	7
Job satisfaction	0,868	5
Stress recognition	0,785	4
Perception of management	0,921	3
Working conditions	0,863	4
SAQ	0,936	29

A normal distribution was not present in Kolmogorov–Smirnov and Shapiro–Wilk-test for all subscales (abnormal in stress recognition and working conditions). Therefore, subgroups were analyzed with the Kruskal–Wallis- as well as the Mann–Whitney-U-test.

The Mann–Whitney-U-test comparing the responses of participants with or without eGENA in their departments showed a significantly higher median SAQ in the departments with eGENA implementation (U (N_(eGENA)_ = 6, N_(non-eGENA)_ = 14) = 70,0, z = 2,31, *p* = 0,02), as well as a large effect size (*r* = 0,516). We therefore performed a Mann–Whitney-U-test for all subscales. The Median eGENA departments was significantly higher with a moderate to large effect in 4 of the 6 subscales (Table 3). Surprisingly the subscale “stress recognition” showed by far the least differences between the subgroups.

**Table 3:** Medians, interquartile ranges and Mann–Whitney-U-tests for eGENA and non-eGENA Departments

	**eGENA Departments**		**non-eGENA Departments**		**Mann–Whitney-U**		
**Scale**	**MD**	**IQR**	**MD**	**IQR**	**U**	**z**	**p**
Teamwork climate	4,01	0,72	2,89	1,40	91,0	2,39	0,016
Safety climate	3,39	1,13	2,77	1,09	80,0	1,94	0,056
Job satisfaction	4,12	0,91	2,99	2,02	83,5	2,19	0,026
Stress recognition	4,13	1,98	4,08	1,03	54,0	-0,39	0,728
Perception of management	3,65	1,89	1,52	2,47	95,5	2,29	0,019
Working conditions	4,24	1,36	2,30	1,74	93,5	2,16	0,028
SAQ	3,74	0,90	2,82	0,77	70,0	2,31	0,020

Because of the small sample size, a Mann–Whitney-U-test for differences by occupation was performed for compiled subgroups of physicians (including consultants and residents; *N* = 14) as well as a subgroup of nurses (including nurses, specialized nurses and anesthesia technicians; *N* = 9) (Table 4). While the median SAQ was significantly higher with a large effect size for physicians only 3 (job satisfaction, perception of management and working conditions) of the 6 subgroups achieved statistical significance, with moderate to large effect sizes.

**Table 4:** Medians, interquartile ranges and Mann–Whitney-U-tests for nurses and physicians

	**Physicians**		**Nurses**		**Mann–Whitney-U**		
**Scale**	MD	IQR	MD	IQR	U	z	p
Teamwork climate	4,01	0,81	3,10	1,73	78,0	1,50	0,145
Safety climate	3,39	1,37	2,93	1,04	73,5	1,00	0,324
Job satisfaction	4,12	0,89	3,44	1,60	84,5	1,74	0,082
Stress recognition	3,79	2,14	4,43	1,43	49,5	-0,85	0,403
Perception of management	3,86	1,64	1,15	2,23	105,5	2,68	0,005
Working conditions	4,32	0,97	2,48	1,61	110,0	2,96	0,002
SAQ	3,78	0,96	2,92	1,37	79,0	2,39	0,031

Kruskal–Wallis tests for differences by gender and relevant work experience did not achieve statistical significance in the SAQ as well as its subscales.

## Discussion

**Fig. 1:**
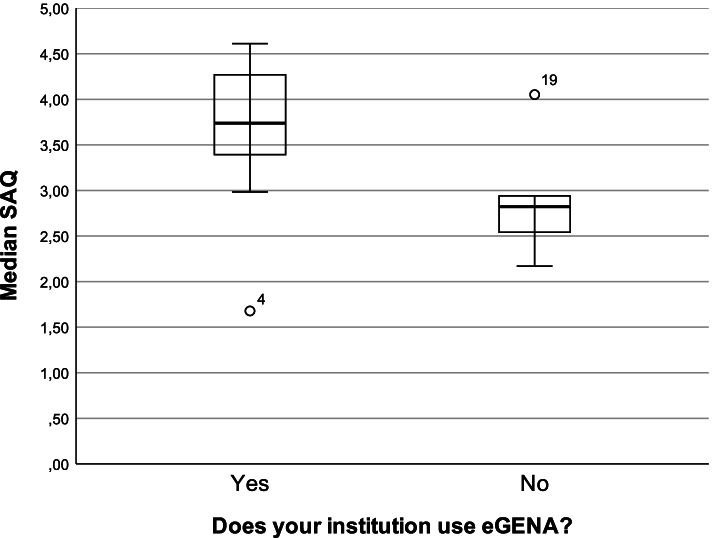
SAQ of eGENA and non-eGENA departments

Statistically significant differences between eGENA and non-eGENA departments and between occupations could be attained although only a small sample size (*n* = 24) could be achieved. Overall participants from eGENA-Departments reported a significantly higher median SAQ-GER than their counterparts from non-eGENA departments indicating a higher SAQ in early adopters of this cognitive aid (Fig. 1). In this context, employees with high SAQ values can be a surrogate for high SAQ values in the entire department [26]. Safety culture is therefore likely to differ significantly from settings where cognitive aids are developed and researched to later adopting settings. This might be one of several reason why, these potent, low-cost tools aren’t used as extensively as their possibilities might allow.

Statistical differences between early and later adopting anaesthesiology departments could be found in the subscales teamwork climate, job satisfaction, perception of management as well as working conditions. But the scale safety climate (U (N_eGENA_ = 6, N_non eGENA_ = 14) = 80,0, z = 1,94, *p* = 0,056, *r* = 0,414) did not achieve statistical significance. Therefore, multiple dimensions of safety culture are different in those early adopting departments. This could lead to extensive effects on the success of cognitive aid implementation. Experiences, insights and best practices from departments that are involved in development and research into cognitive aids therefore most likely must be adapted in different contexts. Since cognitive aids might be cost effective potent tools for improving patient safety the implication of these differences should be investigated further or else these tools might lose their impact and staff participation might fade off. Several strategies and techniques for the increase in safety culture measurements have been published in recent years. We suggest benchmarking the SAQ against a successful implementation to decide on the value of using one of these before cognitive aid implementation.

Surprisingly the subscale stress recognition showed very similar results in both subgroup assessments (U (N_eGENA_ = 6, N_non eGENA_ = 14) = 54,0, z = -0,39, *p* = 0,728, *r* = -0,081). Other studies, that did not focus on one specific specialty, typically reported lower values in this scale. Though this result can be perceived as favorable, since recognition of stress is an important step minimize its negative effects, the reasons for this discrepancy are unclear. The answer might lie in causes specific to the specialty, the departments or the selection of participants. Further research might provide significant insight in improving stress recognition for institutions.

Regardless of the small sample size respondents from different levels of work experience as well as occupations completed the survey. Therefore, we were able to again attain significant differences in perception of safety culture between occupations, suggesting a significant external validity. But due to the small sample size multiple occupations had to be compiled together. Comparing these compiled groups of physicians and nurses significant differences occurred for the SAQ in general, as well as for the scales job satisfaction, perception of management and working conditions. Noticeably these scales all relate to different aspects determined by management. In the German healthcare system physicians and nurses are typically under different management and work different shifts and hours, while typically caring for one patient in a nurse physician team. Additionally, poor working condition of nurses were and still are part of an ongoing debate in German society. Consequently, the differences by occupation might be caused be external effects, that do not influence cognitive aid implementation. These differences were nonetheless measurable in this study. Several studies have shown differences in cognitive aid acceptance in correlation with occupation [27]. We therefore conclude that frameworks for cognitive aid implementation in German anaesthesiology departments should adequately assess and adjust for local circumstances from different occupations.

Though statistically significant differences between the departments as well occupations could be attained, we recognize several limitations to our study. First the sample size of this study was unexpectedly small. This might be explained by specific idiosyncrasies of the German health system where both anesthetists and anaesthesiology nurses through their training co-specialize for intensive care. During the survey period workload in German ICU due to COVID-19 was especially high and relocation of personnel from operating theaters was not uncommon. Another explanation might be the mode of contact for inclusion in the study, since it relied on high motivation from the participating departments to forward the survey. Likewise, the total size of the respective departments is not known. A response rate could therefore not be calculated. This secondly results in the possibility that single departments might be highly overrepresented, resulting in misinterpretation of the effects of the implementation of eGENA on the SAQ. Thirdly this study did not compare the SAQ with the use of eGENA, but only its availability, and therefore only describes the effects of the occurrence of implementation. Since no data was obtained before the implementation the subgroups of the departments might have shown significant differences before the use of eGENA, so the effect of the implementation can’t be assessed with this study. Yet the small sample size might have shown that small subgroups can be sufficient to generate statistically significant differences in the SAQ-GER. This Score therefore might even be of utility for benchmarking of smaller departments or hospitals, as well as ongoing surveillance.

## Conclusion

Despite a small sample size (*n* = 24), this study likely demonstrates that members of departments that recently adopted the cognitive aid eGENA report a significantly higher median SAQ-GER than peers from departments without eGENA implementation, indicating a higher perception of safety culture in early adopters of cognitive aids. Since it has been shown to both be a significant facilitator and hurdle for implementation success, studying best practices from successful applications might not be enough, rather local leaders should carefully inspect requirements for implementation. Frameworks for implementation of cognitive aids should therefore include a reliable assessment of safety culture. For this benchmark from successful introductions might be of high utility.

Furthermore, some specific characteristics of the German language version of the SAQ could be shown. This includes a low Cronbach’s ⍺ for the subscale perception of management. Therefore, results of this and similar surveys should only be carefully compared to other versions of the SAQ. If the version by Zimmermann et al. would be used for benchmark development the item “Management does not knowingly compromise the safety of patients” from the scale perception of management should be excluded. This further complicates benchmarking against international samples.

Additionally, a significant difference in the SAQ-GER subject to the occupation could be demonstrated. Frameworks for cognitive aid implementation should therefore likely include measures to adequately address all occupations and their individual requirements in German anaesthesiology departments. The reasons for and the implications of these differences remain unclear and warrant further investigation.

Small sample sizes might be sufficient to draw significant conclusions about smaller subgroups. Therefore, the SAQ-GER might be used for ongoing surveillance of the safety climate even of smaller departments. Further investigation of the results of the subscale stress recognition in the participating departments might result in significant insights in improving the effects of stress on patient safety.

## Data Availability

The datasets used and/or analysed during the current study are available from the corresponding author on reasonable request.
